# Emergency management in fever clinic during the outbreak of COVID-19: an experience from Zhuhai

**DOI:** 10.1017/S0950268820001764

**Published:** 2020-08-07

**Authors:** H. Jiang, J.W. Liu, N. Ren, R. He, M.Q. Li, Q.C. Dong

**Affiliations:** 1 Operations Department, Zhuhai People's Hospital (Zhuhai hospital affiliated with Jinan University), Zhuhai 519000, China; 2 Faculty of Medicine, Macau University of Science and Technology, Macau 999078, China; 3 Party Committee of the Communist Party of China, Zhuhai People's Hospital (Zhuhai hospital affiliated with Jinan University), Zhuhai 519000, China; 4 Comprehensive Office, Zhuhai People's Hospital (Zhuhai hospital affiliated with Jinan University), Zhuhai 519000, China; 5 Department of Hospital Infection, Zhuhai People's Hospital (Zhuhai hospital affiliated with Jinan University), Zhuhai 519000, China; 6 Out-patient Department, Zhuhai People's Hospital (Zhuhai hospital affiliated with Jinan University), Zhuhai 519000, China

**Keywords:** COVID-19, emergency management, fever clinic, infection prevention and control, SARS-CoV-2

## Abstract

Coronavirus disease 2019 (COVID-19) is a global health threat. A hospital in Zhuhai adopted several measures in Fever Clinic Management (FCM) to respond to the outbreak of COVID-19. FCM has been proved to be effective in preventing nosocomial cross infection. Faced with the emergency, the hospital undertook creative operational steps in relation to the control and spread of COVID-19, with special focuses on physical and administrative layout of buildings, staff training and preventative procedures. The first operational step was to set up triaging stations at all entrances and then complete a standard and qualified fever clinic, which was isolated from the other buildings within our hospital complex. Secondly, the hospital established its human resource reservation for emergency response and the allocation of human resources to ensure strict and standardised training methods through the hospital for all medical staff and ancillary employees. Thirdly, the hospital divided the fever clinic into partitioned areas and adapted a three-level triaging system. The experiences shared in this paper would be of practical help for the facilities that are encountering or will encounter the challenges of COVID-19, i.e. to prevent nosocomial cross infection among patients and physicians.

## Introduction

The spread of severe acute respiratory syndrome coronavirus 2 (SARS-CoV-2) has already caused severe global health consequences. As of 20 May 2020, SARS-CoV-2 had affected over 4970 thousand people and it was associated with more than 320 000 deaths [1]. SARS-CoV-2 infection is highly contagious and transmissible mainly through respiratory droplets and contact routes [2, 3]. SARS-CoV-2 created arduous challenges demanding substantial time and efforts in its control. Based on the experiences learned from the outbreak of SARS in 2003, the principal strategy in relation to the containment and prevention of the spread of Coronavirus disease 2019 (COVID-19) was to build effective fever clinics for triaging patients [4]. The first line of defense against COVID-19 was to establish FCM procedures in high-risk areas of cross-infection. This proved to be the main practical defense in the prevention and control of the spread of COVID-19. How to respond to this epidemic is an essential practical question for comprehensive hospitals to answer.

Located at one of the most prosperous and populous areas of Zhuhai, Zhuhai People's Hospital (the hospital) has provided medical services to many patients from in and out of Zhuhai. In this epidemic, the hospital encountered the first COVID-19 case and screened the largest number of cases in Zhuhai. In Zhuhai, over one million citizens were not local. Many of these non-local residents returned to Zhuhai several days before the outbreak of COVID-19, including many from Hubei [5]. This substantial increase in population augmented the challenges, therefore, our hospital had to encounter in relation to the FCM control, prevention and possible eradication of COVID-19. To achieve its aims and objectives, the hospital decided to redesign and rebuild the fever clinic facility promptly. On 18 January 2020, the hospital began to upgrade the fever clinic facilities and complete the primary construction on the same day to initiate the response as soon as possible. Continuous perfection was performed in the following two months according to actual needs. Additionally, the hospital established a three-level triaging system to identify whether the patients should be transferred to the fever clinic. The FCM operational mechanism had delivered excellent results during the outbreak of COVID-19. Since the completion day on 21 May 2020, a total of 10 436 patients had visited the new fever clinic without causing nosocomial cross infection. During this period, an average of 85 patients visited the fever clinic daily, with a peak of 140 patients in one day. Among these patients, those who had travelled to or lived in the epidemic areas only accounted for 18%, and another 82% was only with clinical symptoms and no travel or living history in the epidemic areas. FCM in the hospital has proven to be a successful operational procedure in the prevention of COVID-19.

## Three operational steps

### Facility upgrading – settings and layout

**Fig. 1.**
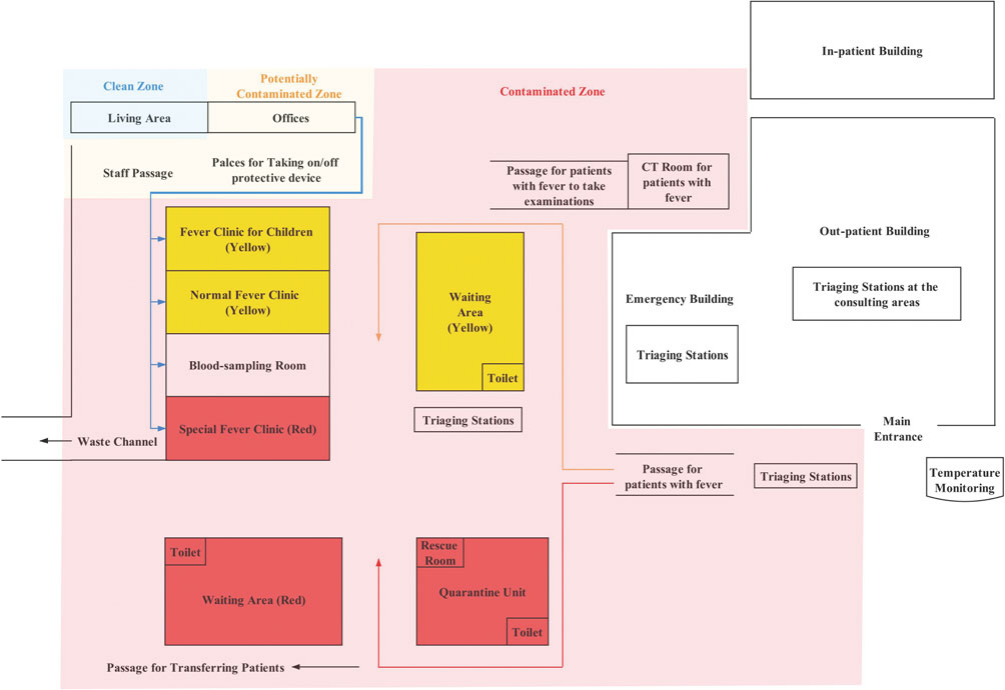
Illustration of the layout and location of fever clinic.

The first operational step is about the settings and layout of the fever clinic. From the view of the hospital, in order to avoid cross infection of COVID-19, the fever clinic should be constructed far away from the other buildings within the hospital complex. With the rapid dissemination of COVID-19, the number of patients had increased dramatically, with the result that previous existing fever clinic procedures were not adequate in effectively and handily administrating the appropriate lifesaving medical procedures. In this circumstance, the hospital recommended that a special treatment area for COVID-19 should be established to control the infection rate of COVID-19. The hospital built several prefab houses to extend and improve the previous two infection clinics, and further increased the treatment infection area to 13 rooms, covering over 700 square metres (as shown in Fig. 1). These 13 rooms are: 1 special fever clinic (red), 2 normal fever clinics (yellow), 1 fever clinic for children, 1 blood sampling room, 1 quarantine unit, 2 waiting areas, 2 offices and 3 special toilets. In Figure 1, the rooms with red colour were set to receive the patients with red identification signs while the rooms with yellow colour were set to receive the patients with yellow identification signs (a detailed description of these two signs is presented in the section “Treatment workflow − partitioned patient management”). A CT room was redesigned to be used only for the purpose of the fever clinic. The upgraded fever clinic was independent of and away from the out-patient and emergency buildings. The revamped infection facilities were divided into three zones: Clean Zone, Potentially Contaminated Zone and Contaminated Zone. These three zones were strictly separated from the rest of the hospital. The passageways in the revamped clinic were divided into three categories: Staff Passage, Patient Passage and Waste Channel. At all time, these three passages were required to be clearly identified. A Quarantine Unit was set up to isolate the patients under observation (one room for each patient) with a rescue room for emergency cases. Triaging stations were scattered at 15 entrances at the first floor and the second floor of the out-patient building, the entrance to the emergency building and the entrance to each ward.

### Human resource management

The second operational step is to manage human resource and provide sufficient training. The hospital initiated an immediate and positive response to the infection of COVID-19 by modernising the Human Resource Management Division. A series of work groups were set up to deal with the challenge presented by the infection of COVID-19, including FCM Expert Group, Triage Workgroup, Transportation Group, Infection Prevention and Control Group and Quality Assurance Group, etc. The upgraded and modernised fever clinic now provides 24/7 services with 14 physicians and 12 nurses working in tandem. To ensure an effective and organised response, the hospital divided the whole day into four periods and endeavoured to arrange each period with sufficient and well-trained healthcare providers. Every day, the healthcare providers concentrated on all appropriated patient care treatments, and used heavy protective equipment. Medical staff received the patients with fever, identified whether they were infected with COVID-19 and assisted the patients with appropriate medical procedures, including blood drawing, sampling and administering the appropriate medical regime. As referenced above, a three-level triaging system was initiated in our hospital with 26 fixed triaging posts and 96 nurses involved in this work.

To provide a safe and adequate medical environment, a training programme was established and performed on a daily basis to respond to the outbreak of COVID-19; that is, all medical staff and their coworkers were required to engage in online and/or offline training courses. For the purpose of continuous improvement in FCM, and to assure a daily scientific response, the hospital carried out supervision of fever clinics and triaging systems. Moreover, the hospital has organised several emergency response drills concerning infection prevention and control, in which medical staff were required to closely cooperate with each other and complete their tasks according to the emergency response plan, including discovering potential cases, initiating the response plan, testing, holding consultations and sterilising tools. These drills aimed at a timely, efficient and organised response to the real emergencies which may suddenly occur. These drills have demonstrated positive results during the outbreak of COVID-19. Medical staff had willingly addressed matters relating to personal protection by wearing masks and performing hand hygiene in a correct and effective way. All wards enjoyed good ventilation, and nosocomial infection had been effectively prevented.

### Triaging system and workflow

#### Three-level triaging system

The third operational step is to set a three-level triaging system and workflows for treating the patients. On-site registration was cancelled and replaced by an online reservation system. As stated early, a three-level triaging system had been adopted including procedures which required all people visiting the hospital to wear surgical masks and take the procedure of body temperature test, detailed inquiry and registration of travel history.

**Level 1:** A triaging station was set outside the entrance to the out-patient building. All people entering the out-patient building must take body temperature test there and show whether they have travelled to or lived in the epidemic areas. The out-patient building at its front door had an intelligent infrared thermometer in position to carry out temperature test more quickly and accurately. Two guide robots with voice broadcast were placed in this area to assist nurses in the primary screening. This screening process had released the work pressure of nurses, and at the same time increased triaging efficiency.

**Level 2:** At the entrance of each treatment area, all of the patients and their families were required to take body temperature test and screen whether they have travelled to or lived in the epidemic areas. After the test and screening, a primary screening form in duplicate would be issued.

**Level 3:** At the guidance desk inside each treatment area, the staff checked whether the primary screening form was correctly completed.

When a patient completed this three-level triaging process, he/she would be directed to different clinics for consultation purpose, and if necessary, appropriate medical treatment pursuant to the triage results. It should be noted that the criteria (including clinical symptoms and epidemiological history) to identify COVID-19 was dynamic, especially for the item ‘Epidemic Regions or High-risk Regions’. The investigative, procedural and operational standards for Level 1, Level 2 and Level 3 were consecutively adjusted according to the epidemic dynamics and risk levels of respective regions both in and outside China. If a patient was identified as a normal patient without symptoms and epidemiological history, he/she would be asked to visit the normal specialist clinics. If the patient was identified as a patient with fever, he/she would be required to visit the fever clinic. All patients must present the primary screening forms to the physician who would check the content and sign on the form if the information was confirmed to be true and correct. After consultation, the physician would retain the first (white) page of the form. The patients and his/her families would take the second (red) page as a certificate. Only with that certificate would the patient be able to take the appropriate medical tests, diagnostic procedures and then be given the relevant medicine and/or receive other appropriate medical procedures.

It should be noted that the above-mentioned processes were mainly for common patients without emergency symptoms. For emergency cases, the processes were simplified to save time for saving life. For emergency cases without fever, if they had no travel or living history in epidemic areas, they were arranged to visit the Emergency Department. For emergency cases without fever but with travel or living history in epidemic areas, and for the emergency cases with fever, there was a green channel for them to get triaged and treated as soon as possible. For those who needed to be immediately rescued like accident victims, the hospital set a rescue room in the Quarantine Unit which was available for treatment and operations.

#### Treatment workflow − Partitioned Patient Management

To prevent and control COVID-19 infection in a timely manner, the hospital pioneered the concept of ‘Partitioned Patient Management’ (PPM). To be more specific, the hospital divided the patients into two groups, which were identified by different marks in the triaging process and set two partitioned waiting areas (red and yellow). **RIS:** For those patients who had travelled to or lived in epidemic areas, and were potentially infected with COVID-19, they were directed to wait in the Waiting Area marked with a Red Identification Sign (RIS). **YIS:** For the patients diagnosed with ‘regular fever’, they were directed to the Waiting Area marked with a Yellow Identification Sign (YIS). All patients transferred to the fever clinic, regardless of their identification signs, were nasopharyngeal swabbed and the collected specimens were analysed; and RT-PCR was then carried out in the rooms of the fever clinic. The samples were then sent to the service desk in the fever clinic, and subsequently transferred by designated staff and vehicles to the appropriate assigned area. The patients with RIS were accompanied by medical staff to the special CT room.

**Fig. 2.**
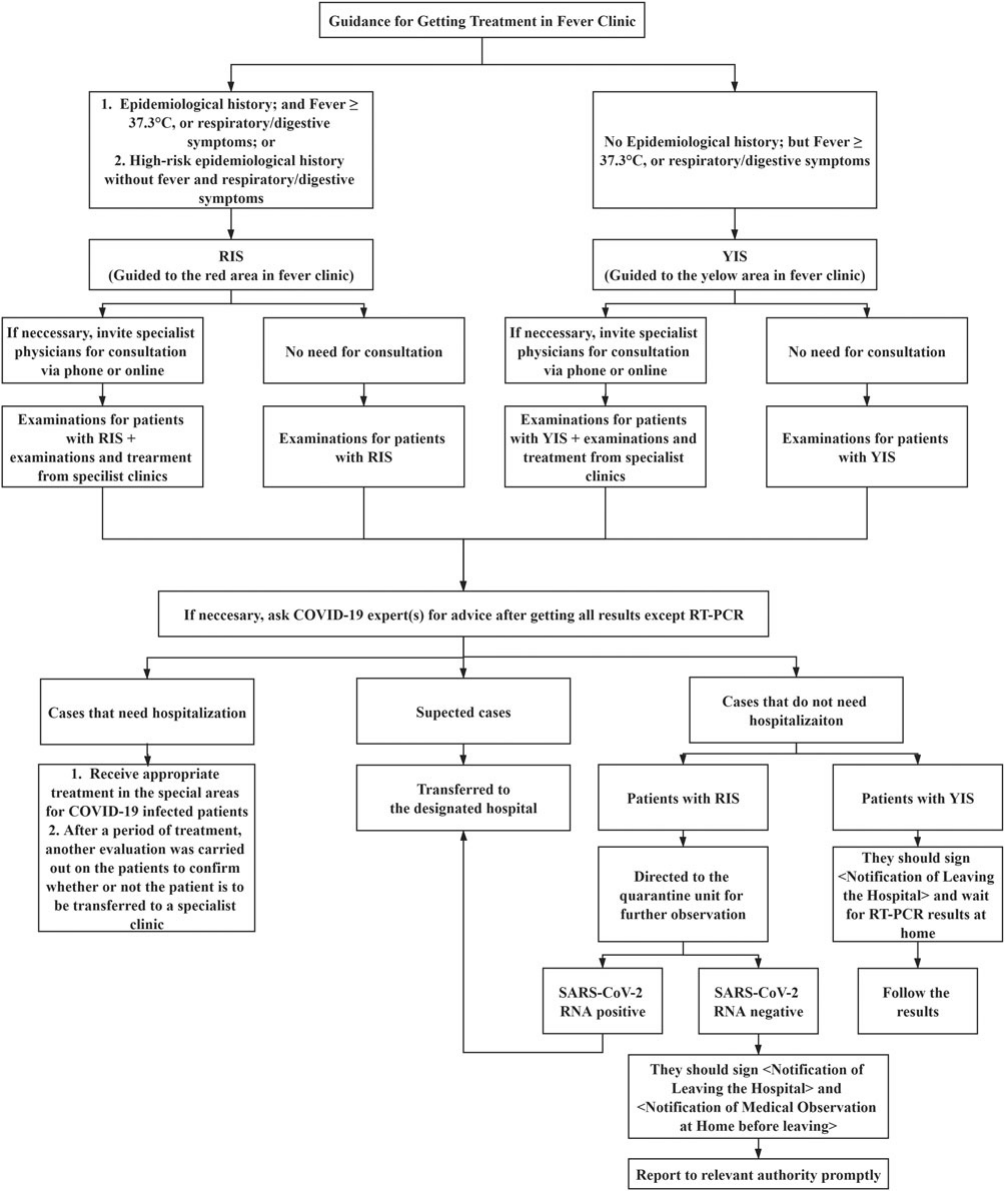
Illustration of treatment workflow in fever clinic.

After completing all procedures, the patients were required to wait for their results in the corresponding waiting area of the fever clinic. Except for RT-PCR, all results were sent to the COVID-19 experts to evaluate the same and then recommend the appropriate medical procedures. Suspected cases were transferred to the designated hospital. The patients who should be hospitalised for treatment would first receive appropriate treatment in the special areas for infected cases. After a period of treatment for COVID-19, another evaluation procedure was carried out on the patients to confirm whether the patients should be transferred to a specialist clinic. Patients with RIS, and found not to require hospitalisation, were directed by medical staff to the Quarantine Unit, where they would get observed and examined. If the patients were tested positive to SARS-CoV-2 RNA, then they would be transferred to the designated hospital. Before leaving the hospital, the patients who tested negative, were required to sign the *Notification of Leaving the Hospital* and *Notification of Medical Observation at Home*. Patients with YIS, and found have no need for to be hospitalised, should sign the *Notification of Leaving the hospital*; and at the same time, they were required to wait in the designated area for the results of RT-PCR. In Figure 2, a flowchart is presented to illustrate this treatment workflow.

The hospital reported fever cases to the relevant authorities every day to establish a closed-loop management. In relation to PPM, the hospital established two kinds of administrative procedures concerning the patients' registration, payment, waiting, treatment, sampling, diagnostic and medical treatment. This PPM operational procedure had in turn, substantially improved the triage efficiency, and significantly prevented nosocomial cross infection. The PPM procedures had obtained the full support and recognition of Zhuhai Healthcare Bureau and the other Zhuhai hospitals.

## Discussion and conclusion

The COVID-19 is an immerging respiratory infectious disease [6]. FCM in the hospital has been very effective in stopping and controlling the spread of coronavirus. As a result of the outbreak of SARS in 2003, many hospitals have become aware of the importance of FCM. During the outbreak of SARS-CoV-2, previous fever clinics were not able to effectively respond to the viral emergency [7].

It is recommended that: (i) A highly reactive, multifunctional and efficient system should be built and administered to respond to the occurrence of COVID-19 infection [8]. (ii) The hospital administration, should make the best use of our space and resources to redesign and rebuild a qualified fever clinic to respond to the COVID-19 outbreak; otherwise, we may not have the capacity to handle future epidemic emergencies. (iii) A rapid human resource management and training programme should be developed as a prerequisite for infection prevention and control. (iv) A standardised fever clinic should be established. (v) Matching treatment procedures should be set up to prevent cross infection. Moreover, after the ending of this epidemic, it is highly suggested that the new facilities of upgraded fever clinic should be reserved and further improved to be permanent buildings available for both daily use and emergency preparation. In regular times, the hospital plans to use those rooms as out-patient clinics (e.g. infectious disease clinics); and change their functions back into fever clinics when another severe epidemic outbreaks unfortunately.

In the current COVID-19 climate, no FCM procedure will suit all situations. As the epidemic continues, corresponding medical and administrative updates and revisions will be necessary to ensure that the inadequacies of previous preventative measures are well addressed by the relevant hospitals and administrative authorities. We trust that this report clearly demonstrates the experiences we have accumulated in the containment and the substantial reduction in the spread of COVID-19.

## Data

The data used to support the findings of this study are available from the authors upon request.
